# Phenylacetic acid metabolic genes are associated with *Mycobacteroides abscessus* dominant circulating clone 1

**DOI:** 10.1128/spectrum.01330-24

**Published:** 2024-09-24

**Authors:** Brittany N. Ross, Emma Evans, Marvin Whiteley

**Affiliations:** 1 School of Biological Sciences and Center for Microbial Dynamics and Infection, Georgia Institute of Technology, Atlanta, Georgia, USA; 2 CF@LANTA-Children’s Cystic Fibrosis Center, Atlanta, Georgia, USA; University of Manitoba, Winnipeg, Manitoba, Canada

**Keywords:** *Mycobacteroides abscessus*, cystic fibrosis, dominant circulating clones, morphotype, phenylacetic acid

## Abstract

*Mycobacteroides abscessus* (MAB) causes lung infections in people with cystic fibrosis (pwCF), and infecting strains show significant genetic variability both between and within individuals. MAB isolates can be divided into dominant clonal clusters (DCCs) or non-clustering groups and can present as smooth or rough colonies on agar plates. Both DCCs and the rough colony morphology have been linked to increased pathogenicity, but the mechanisms are unclear. This study explored the genomes of MAB isolates collected from individuals within the CF@LANTA CF center along with publicly available genomes to identify genes associated with more pathogenic MAB DCCs. Sixty-eight isolates from 26 CF individuals colonized by MAB were morphotyped and sequenced, with almost half of these isolates being members of DCC group 1 (DCC1). While lung function was not significantly impacted by colonization with DCC1 or rough isolates, 102 genes were specifically associated with DCC1 isolates. These genes were enriched for functions in sulfur-based DNA modification, DNA integration, and phenylacetic acid (PAA) catabolism. PAA is produced by the human gut microbiota and found throughout the human body. We show that strains containing PAA metabolic genes allow MAB to use PAA as a sole carbon and energy source. Although the benefits of PAA metabolic genes and other enriched pathways remain unclear, these findings highlight genes associated with emerging MAB CF strains.

**IMPORTANCE:**

A primary challenge in treating bacterial infections is the wide spectrum of disease and genetic variability across bacterial strains. This is particularly evident in *Mycobacteroides abscessus* (MAB), an emerging pathogen affecting people with cystic fibrosis (pwCF). MAB exhibits significant genetic diversity both within and between individuals. However, seven dominant circulating clones (DCCs) have emerged as the major cause of human infections, demonstrating increased pathogenicity. Understanding the mechanisms underlying this increased pathogenicity and the associated genetic factors is crucial for developing novel treatment strategies. Our findings reveal that specific genes are associated with the DCC1 isolate of MAB, many of which are implicated in antimicrobial susceptibility or virulence.

## INTRODUCTION

During infection, microbes adapt to the host environment, sometimes becoming more persistent and pathogenic. This adaptation is evident in bacteria isolated from individuals with chronic infections, such as those in the cystic fibrosis (CF) lung. CF is a genetic disease caused by a mutation in the cystic fibrosis transmembrane conductance regulator (CFTR), an anion channel essential for proper ion balance. Dysfunctional CFTR leads to inflammation and thickened mucus in the lungs, which is poorly cleared, allowing microbes to colonize and cause chronic infections with periodic acute exacerbations (1, 2). In the CF lung, microbes replicate and adapt, occasionally resulting in the emergence of highly persistent and pathogenic strains containing mutations that promote functions critical for long-term colonization and antimicrobial tolerance.

The CF pathogens *Staphylococcus aureus*, (3, 4) *Pseudomonas aeruginosa*, (3, 4) and non-tubulous Mycobacteria (NTMs) (5, 6) can acquire mutations that lead to more pervasive dominant clones or alterations in the bacterial outer surface, some of which result in morphotypic changes of colonies grown on agar plates. While NTMs are the least studied of these CF pathogens, NTM lung infections increased by ~3.5% each year from 2010 to 2019 (7), posing significant challenges for people with CF (pwCF). Infections with the NTM *Mycobacteroides abscessus* (MAB) is of particular concern because it can be found in water samples from domestic premise plumbing (8–10), and once it colonizes the CF lung, it is difficult to eradicate due to intrinsic antibiotic resistance. This results in high rates of treatment failure, which can complicate lung transplantation by colonizing and damaging the new organ (11, 12). MAB comprises three subspecies: *M. abscessus* subsp. abscessus (MABS), *M. abscessus* subsp. massiliense (MMAS), and *M. abscessus* subsp. bolletii (MBOL), with MABS and MMAS being most prominent in CF lung infections (13). Large-scale genome sequencing of pwCF has revealed that most patients are infected with one of seven dominant circulating clones (DCCs). Four DCCs represent MABS strains (DCC1, 2, 4, and 5), while MMAS isolates cluster in to three DCCs (DCC3, 6, and 7). Of the MAB infections in pwCF, 67% of isolates are DCC strains and 91% of those are MABS (DCC1–5) isolates (13). Laboratory data of strains from DCC1 and DCC2 suggest they are more pathogenic (5), likely a result of acquisition of genes encoding proteins involved in lipid metabolism, DNA repair, or susceptibility to nitric oxide and antimicrobials (14).

During long-term infection of the CF lung, MAB can also change from a smooth colony morphology to a rough morphotype. This change to a rough morphotype is associated with clinical pathology, including increased inflammation, development of bronchiectasis, cavitary lesions, reduced lung function, and higher mortality in people with CF. This rough morphotype is conferred by mutations in the biosynthetic or export machinery of a glycopeptidolipid (GPL) found in the outer leaflet of the outer membrane (15, 16). Loss of GPL leads to increased cell hydrophobicity (17), loss of formation of traditional mat biofilms and sliding motility (18), and increased pathology in human cell culture and infection models (19–22).

While phenotypic and genotypic differences between MAB DCCs and the smooth–rough morphotypes continue to be elucidated, questions remain regarding adaptations that are critical for persistence in the CF lung. To address this, we collected isolates from 26 MAB-positive pwCF from the Atlanta Emory Children’s CF center (CF@LANTA) over a 3-year period. We discovered that the genomes of MAB DCC1 strains in the CF@LANTA clinic are enriched for specific genes compared to non-DCC1 strains, including genes encoding proteins critical for the metabolism of phenolics such as phenylacetic acid (PAA).

## RESULTS

### *M. abscessus subsp. abscessus* is dominant in the CF@LANTA population

**Fig 1:**
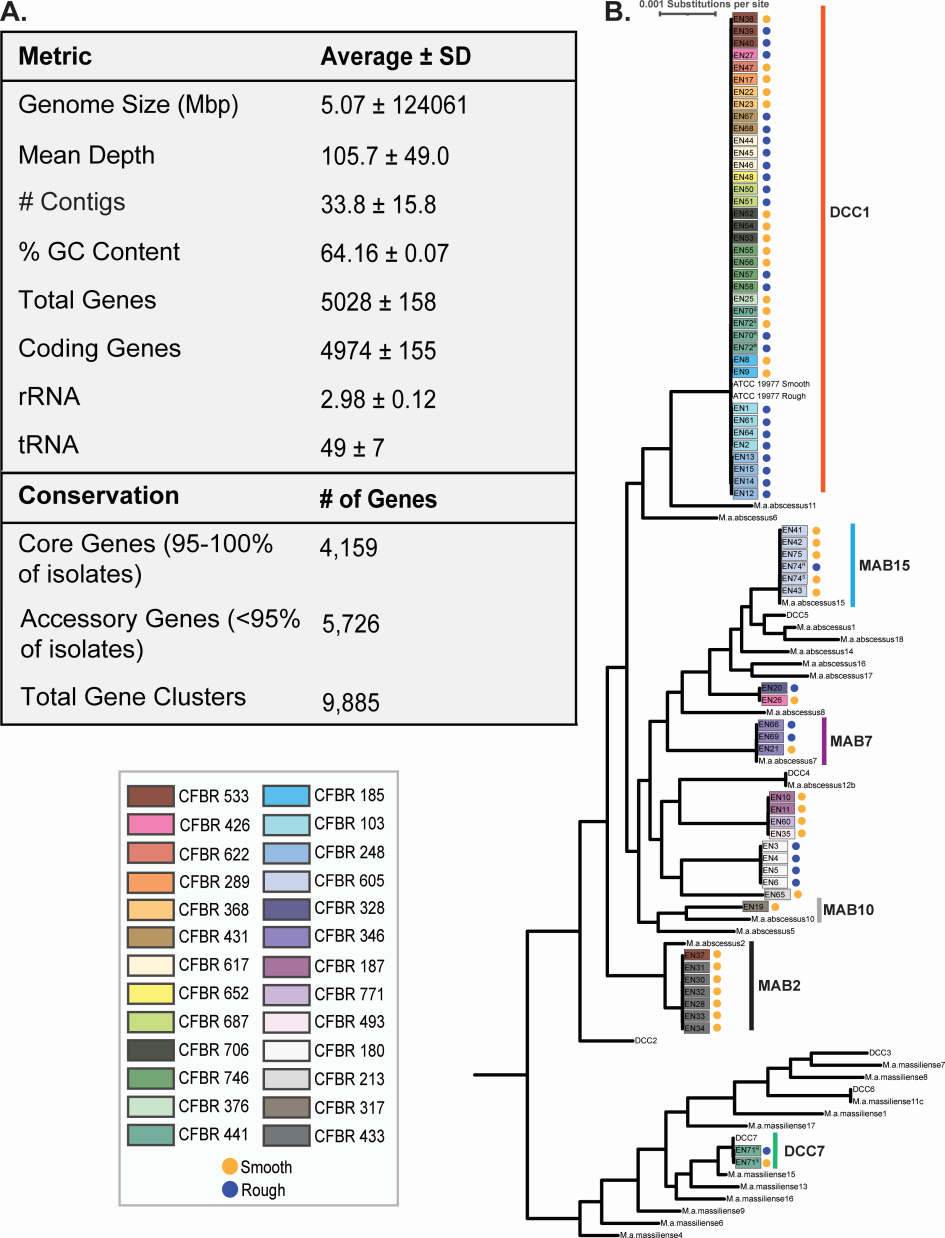
Core genome phylogenetic analysis shows MAB DCC1 is prominent in the CF@LANTA clinic. (**A**) Genome metrics and pangenome results for the 68 isolates sequenced in this study. (**B**) Phylogenic tree generated by alignment of core gene sequences (*n* = 3,913) with strains (designated by EN#) color-coded by participant CF@LANTA biorepository number (CFBR) and colony morphotype. The length of the branches represents the substitutions per site.

To examine the MAB population structure, we first examined patient record data to identify individuals that had a history of being MAB culture-positive between 2019 and 2021 (Table S1). During this time period, 9% (63 out of 700 pwCF) of individuals treated at CF@LANTA were colonized by an NTM. Among these, the most predominant was MAB, accounting for 32 of 63 NTM infections (Fig. S1A). We next collected isolates from these individuals with the goal of culturing MAB for genome sequencing. One to six isolates were obtained from each participant, averaging two to three collections per year (Fig. S1B). Isolates from a total of 68 sputum samples were obtained from 26 of the 32 MAB-positive individuals (Fig. S1B). Single MAB colonies were isolated from each of these sputa originating populations, and the genome was sequenced at an average depth of 105.7 ± 49.0X and an average completeness of 99.98% (Fig. 1A). Metadata from these individuals were also obtained at the time of sputum collection. These data included age, sex, and forced expiratory volume in the first second (pFEV1 Table S1).

**Fig 2:**
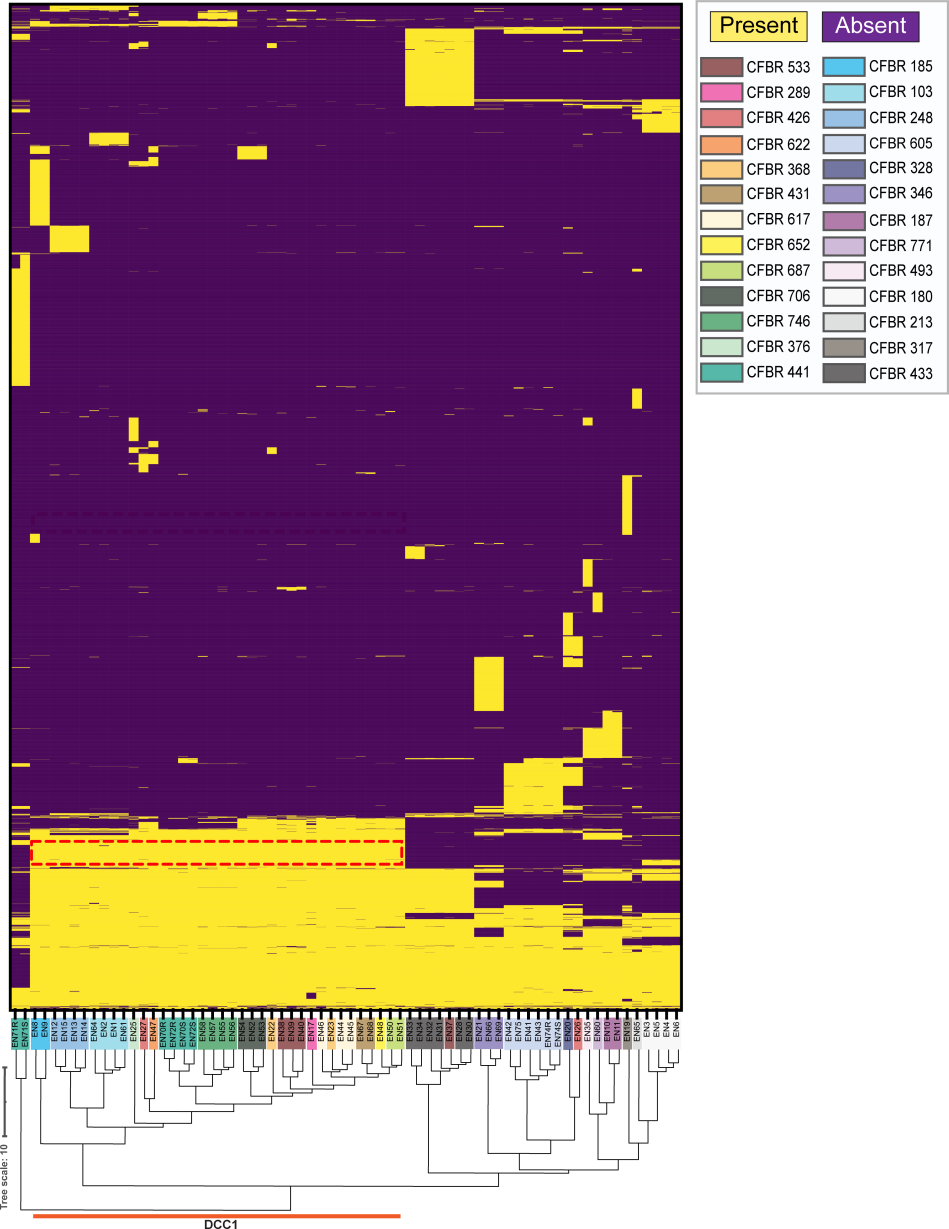
DCC1 isolates have a similar gene content. A heat map depicting the presence (yellow) or absence (purple) of 5,000 MAB genes in CF@LANTA MAB isolates. Each row on the vertical axis represents a gene, and each column on the horizontal axis is an MAB strain (represented by EN#). Strains are color-coded by participant number (CFBR). DCC1 isolates cluster and are indicated by the orange line, and genes present in DCC1 isolates and absent in non-DCC1 isolates are depicted in the red box.

Examination of the 68 MAB genome sequences revealed an average genome size of 5.07 megabase pairs (size range of 4.76 to 5.43 megabase pairs) with an average GC content of 64%. The number of annotated genes ranged from 4,655 to 5,539, with an average gene number of 5,029. Pangenome analysis of these 68 isolates identified 9,885 unique genes, with 4,159 representing core genes (in ≥95% of genomes; Fig. 1A; Table S2). Further pangenome analysis that included an additional 32 publicly available MAB genome sequences (13) (Table S3) increased the pangenome to 16,347 genes and reduced the core genome to 3,913 genes (Table S4). To determine the subspecies and DCC designation of each of the 68 isolates, a phylogenetic tree was generated from the alignment of the core genomes (Fig. 1B). Phylogenetic analysis shows 66 of the strains cluster with MABS with the remaining two isolates, both from one individual, clustering with MMAS in DCC7. Of the 26 pwCF from whom isolates were obtained, 16 (61.5%) are colonized by DCC1 (MABS) isolates, while the remaining 12 participants are colonized by isolates that do not cluster with any DCC (referred to as non-DCC1) (Fig. S1C and D). Note that two individuals were colonized by both DCC1 and non-DCC1 isolates, and an individual colonized by MMAS DCC7 was also colonized by a DCC1 strain. Multi-locus sequence typing (MLST) can also be used to assign MAB strains to DCCs, which was consistent with the phylogenetic clustering (Table S1). Longitudinal isolates from all but three individuals (CFBR 426, CFBR 441, and CFBR 533) clustered together (Fig. 2), indicating that most individuals (*n* = 23) maintain the same strain throughout the sampling period.

In addition to phylogenetic analysis, the colony morphotype on agar plates was noted for each of the 68 isolates. During the study timeframe, twelve participants were continually colonized by the smooth morphotype, eight by the rough morphotype, and six were initially colonized with smooth and transitioned to rough or a mixture of smooth/rough morphotype (Fig. S1C). Overall, 36 smooth and 32 rough isolates were sequenced (Fig. S1D). Previous reports have shown that both DCC1 and rough isolates are more pathogenic, albeit comprehensive clinical correlations are lacking (5, 6, 14, 21, 23, 24). With the CF@LANTA cohort, there was no significant impact of rough or DCC1 colonization on lung function (Fig. S2A through E), although it should be noted that this is a small cohort and that more participants are needed to make definitive conclusions.

### DCC1 genomes have unique gene content

While MAB DCCs arise from the assessment of single-nucleotide polymorphisms (SNPs) in core genes (Fig. 1), similar phylogenic clustering was observed using gene presence/absence (cophenetic correlation coefficient of 0.91, closer to 1 equates to higher similarity, Fig. 2), indicating that the genomes of MAB DCCs have unique gene content. This was not the case with morphotypes as no clustering was evident among rough or smooth isolates. To assess gene content differences in DCCs, we used genome-wide association studies (GWASs), focusing on genes that were present in greater than 80% of strains in one population and less than 20% of strains in the other population using a *P*-value adjusted with Benjamini–Hochberg’s method to correct for the false discovery rate (FDR) of *P* ≤ 0.001.

**Fig 3:**
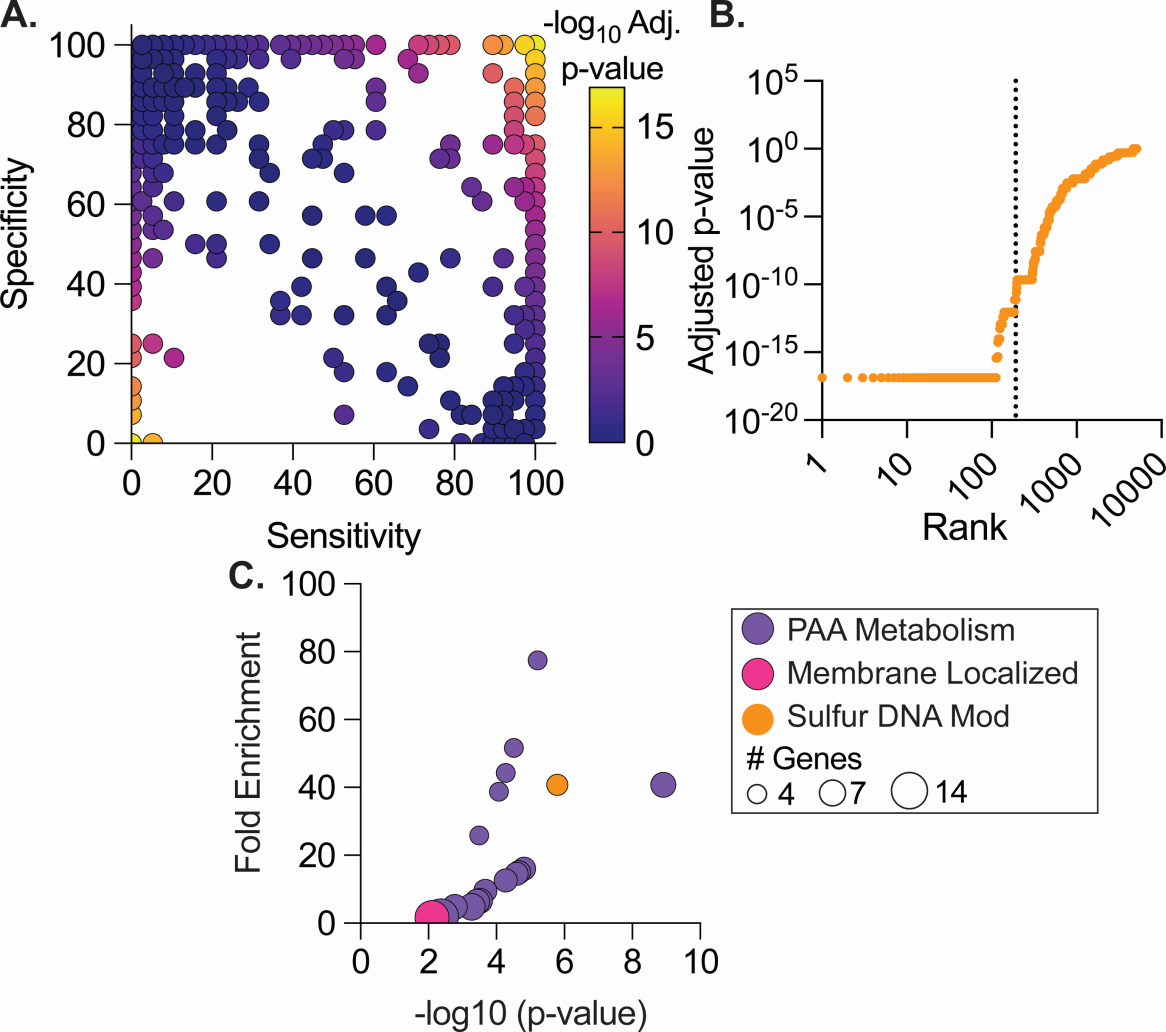
GWAS identifies genes associated with DCC1 and non-DCC1 CF@LANTA MAB isolates. (**A**) Gene sensitivity by specificity showing genes associated with DCC1 (top right) and non-DCC1 (lower left). (**B**) Ranking of MAB genes based on adjusted *P*-value from GWAS analysis. Genes on the left of the vertical line are present in 80% of DCC1 and 20% of non-DCC1 isolates and have a significant adjusted *P*-value. The Benjamini–Hochberg’s method was used to calculate adjusted *P*-values. (**C**) Genes associated with DCC1 isolates were enriched using DAVID and plotted by fold-enrichment and the adjusted *P*-value, color coded by function or localization, and the circle size represents the number of genes enriched.

Stratifying the CF@LANTA MABS genomes by DCC1 vs non-DCC1 revealed 109 genes exclusive to all DCC1 isolates and an additional 80 genes that are highly associated (Fig. 3A; Table S5). These include two large genetic regions MAB_0888–MAB_0918 c and 79.5% of the genes from MAB_1746–MAB_1834 that are significantly associated with DCC1. Conversely, non-DCC1 strains had three exclusive genes and an additional six genes significantly associated with non-DCC1, none of which are present in the DCC1 prototypical strain MAB ATCC 19977 (Fig. 3A; Table S5). These encode 3-oxoacyl-ACP reductase FabG, thiol reductant ABC exporter subunit CydC, septum site-determining protein Ssd, and hypothetical proteins. The differences in the number of genes identified in DCC1 and non-DCC1 strains (189 in DCC1 vs nine in non-DCC1) are likely due to higher genetic diversity of the non-DCC1 group. Functional analysis of the 189 genes specific or highly enriched in DCC1 using Gene Ontology (GO) terms revealed phenylacetic acid (PAA) metabolic genes as highly enriched (*paaF, paaA, paaC, paaK*; 77-fold enrichment, FDR 6e-4; Fig. 3B andC; Table S6). These genes from the PAA pathway were also found in several other enriched pathways involving metabolism of organic acids (Table S6). The *paa* genes (paaZ*, paaR*, *paaJ, paaF, paaH, paaG, paaA-E,* and *paaK*) along with 19 flanking genes (MAB_0888 c–MAB_0918 c; 31,083 bp) are conserved in all DCC1 isolates, except for the gene MAB_0904 c, which was found in 94.7% of isolates (Fig. 3D). Only 14.3% of non-DCC1 isolates carry the *paa* and flanking genes (MAB_0888 c–MAB_0918 c). In addition to the *paa* operon and flanking genes, there was also a significant enrichment of membrane proteins (1.6-fold-enrichment, FDR 0.01) and five genes (MAB_1093 c-1097) involved in DNA modification by sulfur (40-fold-enrichment, FDR 3.7e-5) (25) (Fig. 3C) in DCC1 strains. Comparison to existing data correlating genes to pathogenic phenotypes (26) revealed 25 genes that are important for antibiotic survival, intracellular survival, lethality of *Drosophila melanogaster*, and pyruvate catabolism (Table S5). Finally, a number of phage-associated genes, including 57 genes that are part of an intact phage found in the DCC1 isolate MABS ATCC 19977, were also associated with DCC1 strains.

### Phenylacetic acid is highly conserved and unique to global DCC1 isolates

**Fig 4:**
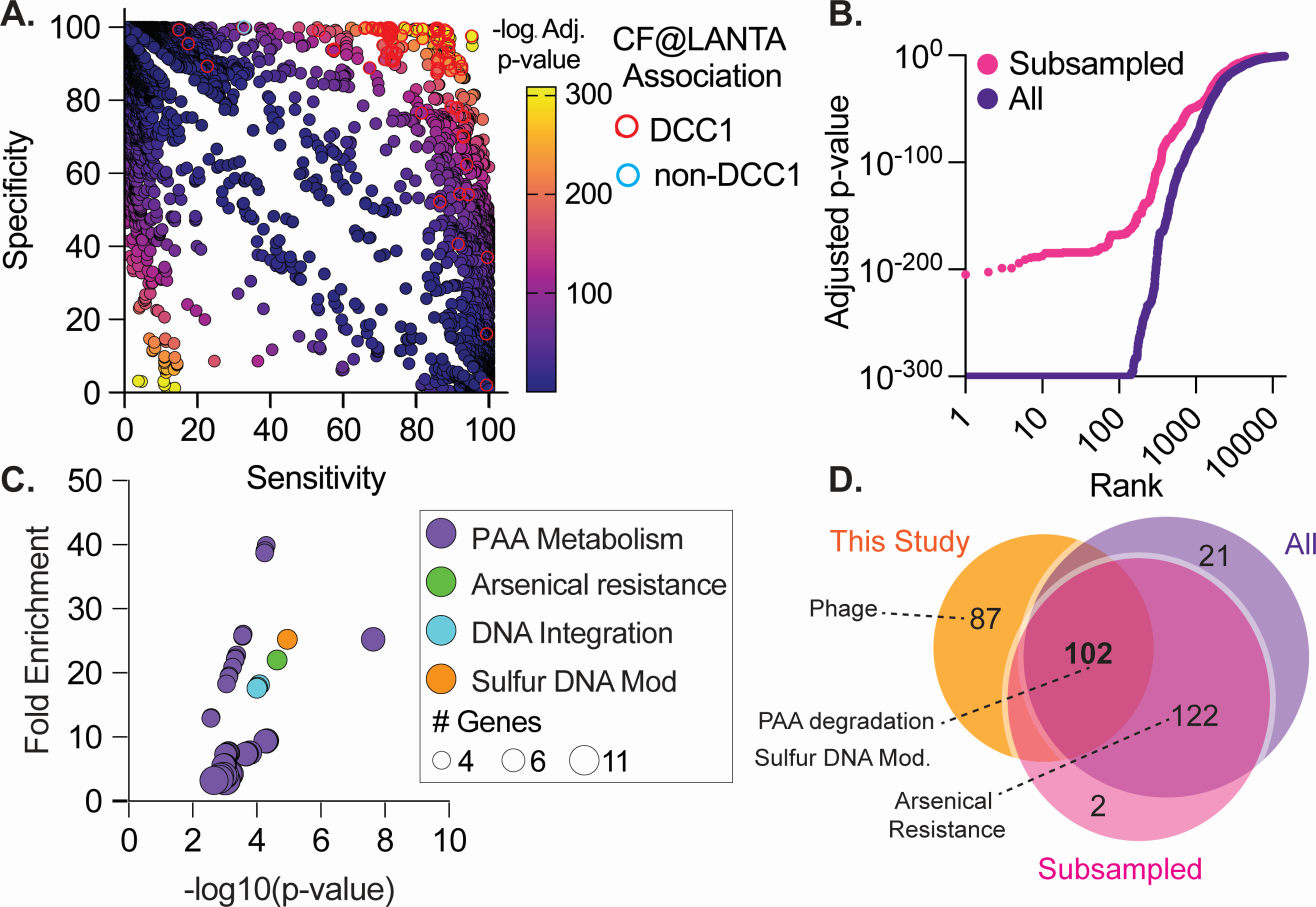
DCC1-associated genes are highly conserved. GWAS was conducted using CF@LANTA MAB genomes as well as an additional 1,964 publicly available genomes. (**A**) Gene sensitivity by specificity showing genes associated with DCC1 (top right) and non-DCC1 (lower left). DCC1 and non-DCC1 unique genes identified with CF@LANTA strains (Fig. 3) are outlined in red and blue, respectively. (**B**) Ranking of MAB genes based on adjusted *P*-value from GWAS analysis. Analysis was performed with all 2,032 MAB genomes (All) as well as equal numbers (460 of each) of DCC1 and non-DCC1 MABS (subsampled). (**C**) Genes associated with DCC1 isolates were enriched using DAVID and plotted by fold-enrichment and the adjusted *P*-value, color-coded by function, and the circle size represents the number of genes enriched. (**D**) Venn diagram of genes enriched in CF@LANTA isolates (CF@LANTA, Fig. 3), all MAB isolates (All), or after subsampling all MAB isolates (subsampled).

To determine if the genes associated with DCC1 strains from CF@LANTA are also observed in other DCC1 strains, publicly available MAB genomes (*n* = 1,964 MABS/MMAS/MBOL; Table S7) were downloaded, and the DCC classification was determined by MLST (Table S8). Following pangenome analysis of these strains, GWAS was carried out using DCC1 (*n* = 460) and non-DCC1 (*n* = 1,572) binary calls (Fig. 4A and B; Table S9). While no single gene was 100% predictive of DCC1 or non-DCC1 groups (Fig. 4A top right and bottom left), GWAS identified 245 genes significantly associated with DCC1 isolates. Included in the genes are the *paa* and 19 flanking genes (MAB_0888 c–MAB_0918 c), as well as genes encoding proteins involved in sulfur-based DNA modification, arsenical resistance, and DNA integration (Table S10).

As the above analysis had different numbers of genomes in each group and included isolates from all MAB subspecies, we further validated our findings by (1) filtering the genomes to include MABS (*n* = 1,406; DCC1 = 460) and (2) subsampled non-DCC1 genomes to 460 in order to match the number of genomes in the DCC1 group. GWAS was then performed on ten subsampled non-DCC1 genome groups (Fig. 4B, pink Table S11). GWAS identified 226 DCC1 enriched genes (lowest adjusted *P*-value 5.5 e-80) and again included enrichment for PAA metabolic genes (44.2-fold-enrichment, FDR 8.9e-4), sulfur-based DNA modification (22-fold-enrichment, FDR 3 = 4.1e-4), arsenical resistance (23-fold-enrichment, FDR 3.1e-4), and DNA integration (20.1-fold-enrichment, FDR 8.9e-4) (Fig. 4C; Table S12). Collectively, these data indicate that 102 genes, including PAA metabolic genes and sulfur-based DNA modification, are enriched in DCC1 strains including those from CF@LANTA (Fig. 4D). In addition, 12 of the 102 DCC1-associated genes have previously been associated with infection-related phenotypes including antibiotic survival, intracellular survival, and lethality of *D. melanogaster* (26) (Table S13). While arsenical resistance was significantly associated with DCC1 when publicly available genomes were included in the analysis, these genes were not included in all three comparisons as they have a higher occurrence among non-DCC1 CF@LANTA isolates (25%–33%Table S5, Fig. 4D).

### The *paa* metabolic genes promote MAB growth on PAA

**Fig 5:**
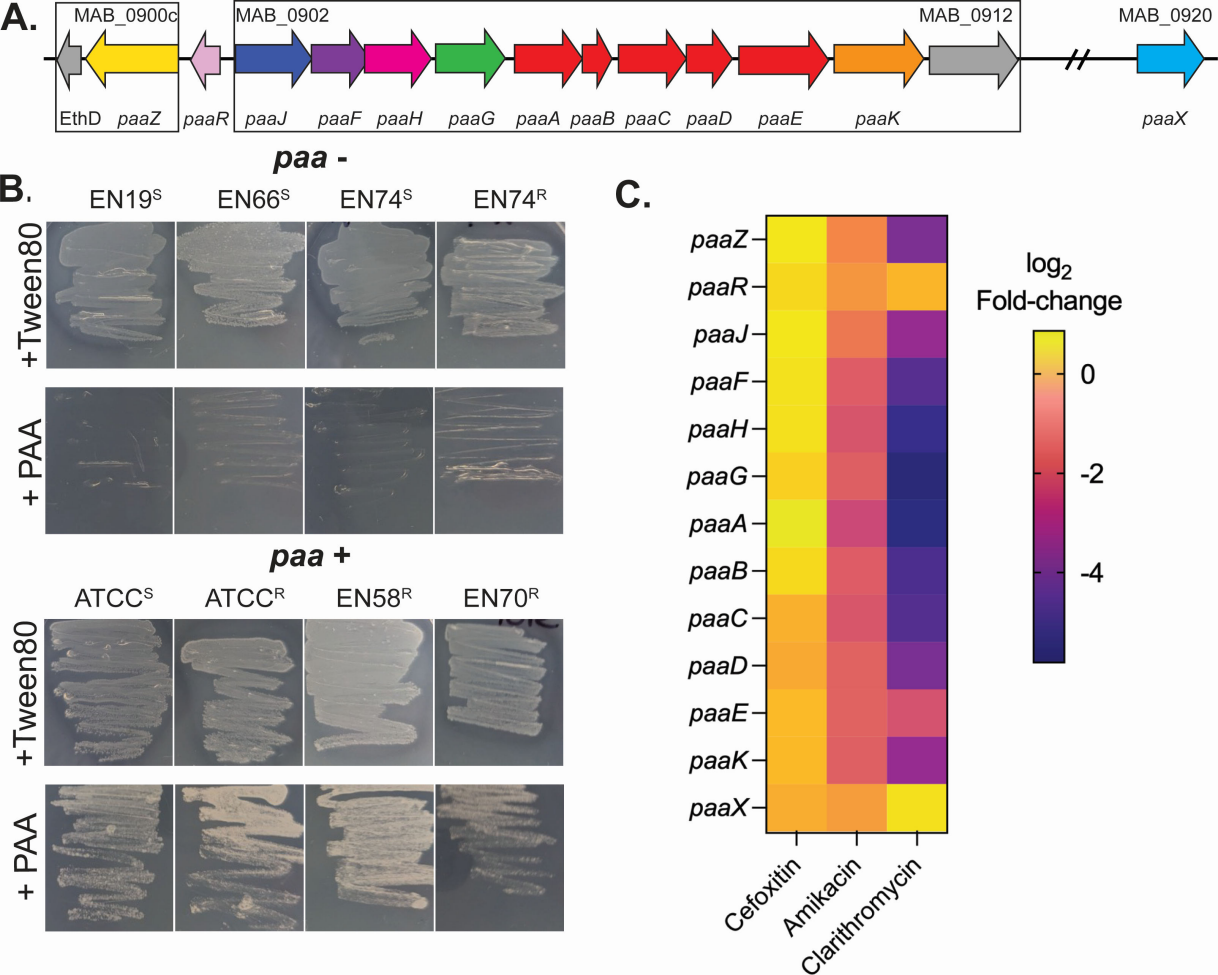
MAB strains containing *paa* genes grow with PAA as the sole source of carbon and energy. (**A**) The MAB DNA region encoding *paa* genes. Putative operons are boxed. (**B**) MAB isolates were grown on MOPS minimal media with either 20 mM Tween-80 or phenylacetic acid (PAA) as the sole carbon and energy source and growth imaged after 96 hours. Representative images are shown. Morphotype, rough (**R**) or smooth (**S**), is denoted by the superscript in the isolate name. (**C**) To investigate paa gene expression upon exposure to antibiotics, publicly available transcriptomes from MAB with and without exposure to amikacin, cefoxitin, and clarithromycin were compared. Shown is the differential gene expression of paa genes, with negative numbers indicating reduced expression upon antibiotic exposure.

The enrichment of genes involved in PAA degradation in DCC1 strains, along with the fact that PAA levels are elevated in the urine of pwCF (27, 28), led us to test if PAA supported the growth of DCC1 and non-DCC1 strains. MABS strains containing *paa* genes grew when PAA was provided as the sole carbon and energy source, while strains lacking these genes did not grow (Fig. 5B). Rather than a carbon source, PAA may also serve as a cue that modulates bacterial physiology (29–35). Previously, genes required for PAA metabolic genes, *paaA, paaB, paaC, paaF,* and *paaK,* were shown to be upregulated in *Acinetobacter baumannii* in response to sub-inhibitory concentrations of antibiotics and promote survival to oxidative stress (31). To test if this also occurred in MAB, publicly available transcriptomes with and without antibiotics were downloaded, reanalyzed, and each sample ranked by the level of expression of the *paa* genes *paaJ, paaF, paaH, paaG, paaA-E,* and *paaK*. A total of 137 MAB transcriptomes contained sequencing reads that mapped to these *paa* genes showing the expression varies 234-fold across conditions (0.0018%–0.04229% TPM; Fig. S3). However, differential expression analysis indicated that exposure to sub-inhibitory cefoxitin in MHB did not impact *paa* expression (Fig. 5C), and sub-inhibitory levels of amikacin and clarithromycin reduced their expression (Fig. 5C). These data indicate that the *paa* genes promote growth of MAB on PAA and that regulation of these genes is environment-dependent.

## DISCUSSION

MAB is an emerging pathogen in pwCF with significant clinical implications, both in regard to therapeutic regimes and overall patient health. We discovered that MAB isolates from our CF@LANTA cohort are members of two dominant clonal clusters, with DCC1 being the most prevalent. These DCC1 isolates have unique genetic content compared to strains from the other DCCs, with the most significantly enriched genes encoding proteins involved in PAA degradation and sulfur-based DNA modification. Genes involved in PAA metabolism have previously been linked to DCC1 isolates (14) and are likely maintained through vertical inheritance evidenced by the high presence in this MAB lineage and syntenic neighboring genes without inverted repeats. PAA is a central intermediate in aromatic hydrocarbon catabolism, degraded to produce acetyl-CoA and succinyl-CoA, which feed into central metabolism (36). It is unclear if humans produce PAA, but gut bacteria can produce PAA as a byproduct of dietary polyphenol breakdown (37), often as an antimicrobial against other colonizing microbes (38–40), and is absorbed into human tissue and excreted in urine (27). While PAA is a uremic toxin, it is detectable in the serum of healthy individuals (~50 µM) and in urine (0.2–4 µM per µM of creatine) (38). While the levels of PAA in the CF lung and in the serum of pwCF are not known, the presence of higher levels of PAA in their urine (27, 28) suggests that PAA may be present at elevated levels throughout the body (39). Thus, MAB may be exposed to elevated PAA in the CF lung, potentially impacting infection outcomes and human health.

The CF lung environment is nutrient-rich, and while PAA or other phenolic compounds may be degraded by this pathway, we hypothesize it is not a primary source of carbon. While we demonstrate that PAA can serve as a carbon and energy source for MABS, in other bacteria, PAA can also act as a cue augmenting bacterial physiology including antimicrobial resistance (29–31), biofilm formation (31), quorum sensing (32, 33), virulence gene expression (34), and immune evasion (31, 35). Contrary to data in other bacteria (29), MAB downregulates *paa* genes in response to frontline therapies amikacin and clarithromycin. If PAA is a cue, the downregulation of catabolic genes may help MAB survive these translation-inhibiting antibiotics. Although *paa* genes have been linked to virulence, we found no significant impact on lung function from DCC1 colonization. A confounding variable is that our study was concurrent to the introduction of highly effective modulator treatment, which has greatly improved the lives of pwCF (40). These findings suggest *paa* genes are maintained in DCC1 isolates, but the exact impact of PAA on physiology requires further research.

DCC1 isolates also exhibit enrichment for DNA integration, sulfur-based DNA modification, and arsenical resistance. Often, organisms undergo reductive evolution to adapt to a particular niche, but DCC1s have many genes that other MAB isolates do not (14). A recent study showed all DCCs have reduced mutation rates (41). The presence of genes encoding proteins critical for DNA integration in DCC1s may compensate for this reduction in mutation frequency by incorporating new genes, aiding MAB adaptation to the CF lung environment. Additionally, sulfur-based DNA modification or DNA phosphonothioate modification was identified. This DNA phosphonothioate involves swapping an oxygen atom for a sulfur atom in the backbone of DNA. While this modification is more susceptible to peracid-mediated, oxidative and amine-catalyzed DNA cleavage in vitro (25), it protects from DNA oxidative stress and promotes survival in a diverse array of stressful environments (42), suggesting a role in promoting survival in the host. Arsenical resistance was also identified in DCC1 isolates and conferred by redundant arsenical reductase genes (MAB_4842 and MAB_4964) and upstream putative ArsR family transcriptional regulators (MAB_4841, MAB_4961 c, and MAB_4962). Arsenic can be found at low levels in drinking water and has been shown to suppress CFTR expression (43), promote lysosomal breakdown of the protein (44), alter toll-like receptor expression (45), and increase sweat chloride, an indicator of CF disease (46).

Several of the DCC1-associated genes identified by GWAS have been implicated as important for survival in infection-relevant conditions and provide new insights into the mechanisms controlling MAB pathogenesis. For example, two genes identified here are important for virulence in a *D. melanogaster* model (MAB_1284 c; MAB_1830) (26), and MAB_0830 affects intracellular survival in macrophages (26), which may explain higher macrophage uptake and increased virulence of DCC1 isolates in SCID mice (5). Thus, leveraging GWAS has allowed for the identification of genes and pathways associated with DCC1 isolates, many of which have been linked to virulence and antimicrobial exposure. With further study, these genes and pathways may serve as drug targets that can reduce the burden and disease severity of MAB infection caused by DCC1 isolates.

## MATERIALS AND METHODS

### Sample source and storage of metadata

Samples and metadata collected for this study were approved by the IRB (H18220). Isolates originated from sputum or BAL samples were collected in the clinic and sent to the CF@LANTA clinical lab. Samples were processed by decontamination to reduced non-*Mycobacterial* species, grown in a nonradiometric *mycobacteria* growth indicator *tube* (MGIT), and provided as streaks on Lowenstein–Jensen agar slants. Metadata associated with the clinic visit were collected and deidentified prior to being received. Data were maintained on a secure server.

### Bacterial strain isolation, growth, and storage

Upon receipt of Lowenstein–Jensen agar slants from the clinical lab, bacterial populations were streaked on 7H11 agar and incubated for 5–7 days. One or more representative colonies were cultured in 7H9 broth supplemented with glycerol, OADC, and 0.05% Tween-80 for 3 days and stocked. Stocks were stored in 12% glycerol at −80 ˚C. Morphotyping was done by visual examination on the primary streak plate. If multiple colony morphotypes were present, they were isolated and stocked. When used, isolates were streaked from freezer stocks on 7H11, grown for 3–5 days at 37°C, and then cultured in 7H9 broth supplemented with glycerol, OADC, and 0.05% Tween-80 for 3 days. For growth on MOPS minimal agar, the base media was prepared (50 mM MOPS, 43 mM NaCl, 93 mM NH_4_Cl, and 2 mM KH_2_PO_4_), pH adjusted to 7.2, and agar added at 15 g/L prior to autoclaving. After cooling, the media was supplemented with 1 µL/mL FeSO_4_, 1 mM MgSO_4_, and 10 µM Tween-80 or phenylacetic acid. Isolates were streaked on MOPS agar from freezer stocks and liquid cultures and grown for 5 days.

### Whole-genome sequencing

Isolates were grown from a single colony in 10 mL of supplemented 7H9. Cultures were centrifuged and DNA isolated using the MasterPure Gram Positive DNA Purification kit (Lucigen) with modifications. Briefly, bacteria were resuspended in 185 µL TE, transferred to lysis matrix B tubes, and 2 µL of lysozyme was added and incubated for 2 hours at 37°C. The GP lysis solution was added at 300 µL per sample and tubed bead-beat three times for 45 seconds each, placing the samples on ice to cool after each bead beating interval. Next, 2 µL of proteinase K was added, heated to 65°C–70°C for 15 minutes, and then incubated at 55°C for 2 hours. Samples were cooled to 37°C, after which 350 µL of MPC protein and vortexed. Debris was pelleted by centrifugation at 4°C for 10 minutes at >10,000 x *g*. To further remove the debris, the supernatant was transferred to a new tube and recentrifuged before transferring to a fresh tube. Samples were depleted of RNA using 2 µL of RNaseA and incubating at 37°C for 30 minutes. The resulting DNA was precipitated by adding 700 µL of isopropanol inverting to mix, centrifuging for 10 minutes at 10,000 rpm at 4°C. Isopropanol was removed, and the DNA pellet washed twice with 70% ethanol, dried, and resuspended in 100 µL of dH2O. DNA integrity was validating by running on an agarose gel and concentration quantified by Qubit. DNA samples were sent to the Microbial Sequencing and Analysis Center (MIGS, Pittsburg, USA) or SeqCoast (New Hampshire, USA) for sequencing. DNA libraries were generated using the Illumina DNA Prep kit and custom IDT 10-bp unique dual indices with a target insert size of 320 bp. Sequencing was performed on an Illumina NovaSeq 6000 sequencer in one or more multiplexed shared-flow-cell runs, producing 2 × 151 bp paired-end reads.

### Genome assembly and analysis

Genome reads were *de novo* assembled using Bactopia v3.0.0 using the flags—shovill_assembler spades –species “Mycobacteroides abscessus.” Assemblies were annotated using Prokka and MLST-typed via PubMLST, all housed in the Bactopia pipeline. To calculate the depth of coverage, the original reads were mapped to their respective assembly using BBMap (2010), which contains pileup.sh. Genome completeness and level of contamination were calculated by CheckM v1.0.18. A pangenome was generated from all genomes with 99% or better completeness and contamination <1% using Bactopia with the flag –use-roary (cluster if ≥95% sequence identity). Core genes were defined as those present in 95% or more genomes. Bactopia automatically passes the core alignment to the IQ tree with the default parameters of Type = string; bootstrap replicates = 1,000. The core-genome.iqtree was visualized and exported from iTol (https://itol.embl.de/), and the CF biorepository number (CFBR) that corresponds to the individual CF the isolates had originated from was added using Adobe Illustrator. To generate a phylogenic tree based on gene presence/absence, the Roary gene_presence_absence.csv output file was uploaded into R, converted to a binary format, and genes and isolates clustered using hclust, method= Euclidean, available in the hclust1d v0.1.1 package. A phylogenic tree was generated using Ape v5-7.1 and visualized and exported from iTol (https://itol.embl.de/). The binary formatted data were plotted as a heat map in GraphPad Prism v10, and the phylogenetic trees were aligned to generate one image. To determine the similarity between isolate clustering by core genes and by gene presence and absence, the cophenetic correlation coefficient was calculated using the dendextend v1.17.1 R package.

For analysis using publicly available genomes used as representative isolates in the phylogenic tree (Table S1) (13), raw read files were downloaded and treated in the same fashion as the genome sequenced here, as described in the above paragraph. Genomes solely used for GWAS were downloaded, in genome or assembly form, from NCBI and reannotated using Prokka via Bactopia.

### GWAS

For genome-wide association (GWAS), isolates were binary-classified based on being DCC1 (1) / non-DCC1 (0). Scoary v1.6.16 was used with 10,000 iterations, flag -e 10000 (47). Genes were filtered by native *P*-value ≤ 0.05, Benjamini and Hochberg adjusted *P*-value ≤ 0.001, and false discovery rate of ≤ 0.001. DCC1 vs non-DCC1 genes were further filtered by gene presence in greater than 80% of one and less than 20% in the opposing cluster type. For enrichment, genes were uploaded to David using UniProt IDs. Enriched pathways were filtered on native *P*-value ≤0.05, Benjamini and Hochberg adjusted *P*-value ≤ 0.05, and false discovery rate of ≤ 0.05. Clustering of pathway was done using the default DAVID settings to enhance the understanding and provide a color code for the data presented (48). Enrichment was also verified using FUNAGE-Pro (49). Phage analysis in ATCC 19977 was done by PHASTER and proteins identified in this study compared using the ATCC 19977 locus IDs (50).

### Transcriptomic analysis

Available transcriptomic data were pulled from European Nucleotide Archive. All data were analyzed using the pipeline regardless of whether they are *in vitro* or *in vivo*. Briefly, reads smaller than 22 bp were removed (cutadapt v3.3), mapped (bowtie2 v2.4.2) to the human genome to remove reads for tissue culture or *in vivo* samples, and to decoy removes reads that may be present from other organisms. Reads that did not map to the human or decoy.fna were mapped to MAB ATCC 19977 and reads tallied using featureCounts v1.6.3. Only samples that had reads mapping to ≥3,000 CDS were included in downstream analysis. To determine the expression across all samples, raw data were transformed to transcripts per million and percent of all transcripts calculated per gene. The percent of reads that map to the region encoding the *paa* operons was obtained from summing the %TPM of the main enzymatic operon MAB_0902–0912, and only samples with expression of 10 or more of the 11 genes were included. Differential expression analysis was done with DESeq2 v.1.40.1 with a significance cut of *P* ≤ 0.05. The code is available at https://github.com/brross60/RNAseq/blob/main/trim_map_invivo.sh.

## Data Availability

All isolates sequenced in this publication are available at PRJNA1119444. A list of all samples used in this work can be found in Table S1.

## SUPPLEMENTAL MATERIAL

Figures S1 to S3 spectrum.01330-24-s0001.docx Fig. S1: Emory MAB population and Clinical Isolate Collection. Fig. S2: MAB DCC and Morphotype Colonization and Association with Lung Function. Fig. S3: *paa* Expression across publicly-available transcriptomes.

Tables S1 and S2 spectrum.01330-24-s0002.xlsx CF@TLANTA Isolates and Pangenome of CF@LANTA isolates.

Tables S3 and S4 spectrum.01330-24-s0003.xlsx Table S3: DCC and Non-DCC Representative MAB Isolates. Table S4: Pangenome of isolates sequenced here and representative isolates.

Tables S5 and S6 spectrum.01330-24-s0004.xlsx Table S5: Genes associated to CF@LANTA DCC1 isolates as defined by GWAS. Table S6: Enrichment of DCC1 associated genes from CF@LANTA isolates.

Tables S7 to S10 spectrum.01330-24-s0005.xlsx Table S7: Publicly Available MAB Isolates. Table S8: MLST Typing of MAB Isolates. Table S9: Genes associated to DCC1 isolates from all isolated as defined by GWAS. Table S10: Enrichment of DCC1 associated genes.

Tables S11 to S13 spectrum.01330-24-s0006.xlsx Table S11: Genes associated to DCC1 isolates. Table S12: Enrichment of DCC1 associated genes. Table S13: Function and fitness association of genes conserved among analyses.

ASM does not own the copyrights to Supplemental Material that may be linked to, or accessed through, an article. The authors have granted ASM a non-exclusive, world-wide license to publish the Supplemental Material files. Please contact the corresponding author directly for reuse.

## References

[1] De Boeck K. 2020. Cystic fibrosis in the year 2020: a disease with A new face. Acta Paediatr 109:893–899. doi:10.1111/apa.15155 31899933

[2] Turcios NL. 2020. Cystic fibrosis lung disease: an overview. Respir Care 65:233–251. doi:10.4187/respcare.06697 31772069

[3] Millette G, Séguin DL, Isabelle C, Chamberland S, Lucier J-F, Rodrigue S, Cantin AM, Malouin F. 2023. Staphylococcus aureus small-colony variants from airways of adult cystic fibrosis patients as precursors of adaptive antibiotic-resistant mutations. Antibiotics (Basel) 12:1069. doi:10.3390/antibiotics12061069 37370388 PMC10294822

[4] Nagashima H, Takao A, Maeda N. 1999. Abscess forming ability of Streptococcus milleri group: synergistic effect with Fusobacterium nucleatum. Microbiol Immunol 43:207–216. doi:10.1111/j.1348-0421.1999.tb02395.x 10338189

[5] Bryant JM, Grogono DM, Rodriguez-Rincon D, Everall I, Brown KP, Moreno P, Verma D, Hill E, Drijkoningen J, Gilligan P, et al.. 2016. Emergence and spread of a human-transmissible multidrug-resistant nontuberculous mycobacterium. Science 354:751–757. doi:10.1126/science.aaf8156 27846606 PMC5142603

[6] Catherinot E, Roux A-L, Macheras E, Hubert D, Matmar M, Dannhoffer L, Chinet T, Morand P, Poyart C, Heym B, Rottman M, Gaillard J-L, Herrmann J-L. 2009. Acute respiratory failure involving an R variant of Mycobacterium abscessus. J Clin Microbiol 47:271–274. doi:10.1128/JCM.01478-08 19020061 PMC2620830

[7] Marshall JE, Mercaldo RA, Lipner EM, Prevots DR. 2023. Incidence of nontuberculous mycobacteria infections among persons with cystic fibrosis in the United States (2010-2019). BMC Infect Dis 23:489. doi:10.1186/s12879-023-08468-6 37488500 PMC10364346

[8] Chadha R, Grover M, Sharma A, Lakshmy A, Deb M, Kumar A, Mehta G. 1998. An outbreak of post-surgical wound infections due to Mycobacterium abscessus. Pediatr Surg Int 13:406–410. doi:10.1007/s003830050350 9639628

[9] Baker AW, Lewis SS, Alexander BD, Chen LF, Wallace RJ, Brown-Elliott BA, Isaacs PJ, Pickett LC, Patel CB, Smith PK, et al.. 2017. Two-phase hospital-associated outbreak of Mycobacterium abscessus: investigation and mitigation. Clin Infect Dis 64:902–911. doi:10.1093/cid/ciw877 28077517 PMC5848312

[10] Thomson R, Tolson C, Sidjabat H, Huygens F, Hargreaves M. 2013. Mycobacterium abscessus isolated from municipal water - a potential source of human infection. BMC Infect Dis 13:241. doi:10.1186/1471-2334-13-241 23705674 PMC3668184

[11] Nessar R, Cambau E, Reyrat JM, Murray A, Gicquel B. 2012. Mycobacterium abscessus: a new antibiotic nightmare. J Antimicrob Chemother 67:810–818. doi:10.1093/jac/dkr578 22290346

[12] Pasipanodya JG, Ogbonna D, Ferro BE, Magombedze G, Srivastava S, Deshpande D, Gumbo T. 2017. Systematic review and meta-analyses of the effect of chemotherapy on pulmonary Mycobacterium abscessus outcomes and disease recurrence. Antimicrob Agents Chemother 61:e01206-17. doi:10.1128/AAC.01206-17 28807911 PMC5655093

[13] Ruis C, Bryant JM, Bell SC, Thomson R, Davidson RM, Hasan NA, van Ingen J, Strong M, Floto RA, Parkhill J. 2021. Dissemination of Mycobacterium abscessus via global transmission networks. Nat Microbiol 6:1279–1288. doi:10.1038/s41564-021-00963-3 34545208 PMC8478660

[14] Bryant JM, Brown KP, Burbaud S, Everall I, Belardinelli JM, Rodriguez-Rincon D, Grogono DM, Peterson CM, Verma D, Evans IE, Ruis C, Weimann A, Arora D, Malhotra S, Bannerman B, Passemar C, Templeton K, MacGregor G, Jiwa K, Fisher AJ, Blundell TL, Ordway DJ, Jackson M, Parkhill J, Floto RA. 2021. Stepwise pathogenic evolution of Mycobacterium abscessus. Science 372:eabb8699. doi:10.1126/science.abb8699 33926925 PMC7611193

[15] Park IK, Hsu AP, Tettelin H, Shallom SJ, Drake SK, Ding L, Wu U-I, Adamo N, Prevots DR, Olivier KN, Holland SM, Sampaio EP, Zelazny AM. 2015. Clonal diversification and changes in lipid traits and colony morphology in Mycobacterium abscessus clinical isolates. J Clin Microbiol 53:3438–3447. doi:10.1128/JCM.02015-15 26292297 PMC4609687

[16] Pawlik A, Garnier G, Orgeur M, Tong P, Lohan A, Le Chevalier F, Sapriel G, Roux A-L, Conlon K, Honoré N, Dillies M-A, Ma L, Bouchier C, Coppée J-Y, Gaillard J-L, Gordon SV, Loftus B, Brosch R, Herrmann JL. 2013. Identification and characterization of the genetic changes responsible for the characteristic smooth-to-rough morphotype alterations of clinically persistent Mycobacterium abscessus. Mol Microbiol 90:612–629. doi:10.1111/mmi.12387 23998761

[17] Viljoen A, Viela F, Kremer L, Dufrêne YF. 2020. Fast chemical force microscopy demonstrates that glycopeptidolipids define nanodomains of varying hydrophobicity on mycobacteria. Nanoscale Horiz 5:944–953. doi:10.1039/c9nh00736a 32314749

[18] Ryan K, Byrd TF. 2018. Mycobacterium abscessus: shapeshifter of the mycobacterial world. Front Microbiol 9:2642. doi:10.3389/fmicb.2018.02642 30443245 PMC6221961

[19] Kam JY, Hortle E, Krogman E, Warner SE, Wright K, Luo K, Cheng T, Manuneedhi Cholan P, Kikuchi K, Triccas JA, Britton WJ, Johansen MD, Kremer L, Oehlers SH. 2022. Rough and smooth variants of Mycobacterium abscessus are differentially controlled by host immunity during chronic infection of adult zebrafish. Nat Commun 13:952. doi:10.1038/s41467-022-28638-5 35177649 PMC8854618

[20] Catherinot E, Clarissou J, Etienne G, Ripoll F, Emile J-F, Daffé M, Perronne C, Soudais C, Gaillard J-L, Rottman M. 2007. Hypervirulence of a rough variant of the Mycobacterium abscessus type strain. Infect Immun 75:1055–1058. doi:10.1128/IAI.00835-06 17145951 PMC1828507

[21] Howard ST, Rhoades E, Recht J, Pang X, Alsup A, Kolter R, Lyons CR, Byrd TF. 2006. Spontaneous reversion of Mycobacterium abscessus from a smooth to a rough morphotype is associated with reduced expression of glycopeptidolipid and reacquisition of an invasive phenotype. Microbiol (Reading) 152:1581–1590. doi:10.1099/mic.0.28625-0 16735722

[22] Caverly LJ, Caceres SM, Fratelli C, Happoldt C, Kidwell KM, Malcolm KC, Nick JA, Nichols DP. 2015. Mycobacterium abscessus morphotype comparison in a murine model. PLoS One 10:e0117657. doi:10.1371/journal.pone.0117657 25675351 PMC4326282

[23] Kreutzfeldt KM, McAdam PR, Claxton P, Holmes A, Seagar AL, Laurenson IF, Fitzgerald JR. 2013. Molecular longitudinal tracking of Mycobacterium abscessus spp. during chronic infection of the human lung. PLoS One 8:e63237. doi:10.1371/journal.pone.0063237 23696800 PMC3655965

[24] Hedin W, Fröberg G, Fredman K, Chryssanthou E, Selmeryd I, Gillman A, Orsini L, Runold M, Jönsson B, Schön T, Davies Forsman L. 2023. A rough colony morphology of Mycobacterium abscessus is associated with cavitary pulmonary disease and poor clinical outcome. J Infect Dis 227:820–827. doi:10.1093/infdis/jiad007 36637124 PMC10043986

[25] Zhou X, He X, Liang J, Li A, Xu T, Kieser T, Helmann JD, Deng Z. 2005. A novel DNA modification by sulphur. Mol Microbiol 57:1428–1438. doi:10.1111/j.1365-2958.2005.04764.x 16102010

[26] Boeck L, Burbaud S, Skwark M, Pearson WH, Sangen J, Weimann A, Everall I, Bryant JM, Malhotra S, Bannerman BP, Kierdorf K, Blundell TL, Dionne MS, Parkhill J, Floto RA. 2021. The pathobiology of Mycobacterium abscessus revealed through phenogenomic analysis. bioRxiv. doi:10.1101/2021.10.18.464689

[27] Seakins JW. 1971. The determination of urinary phenylacetylglutamine as phenylacetic acid. Studies on its origin in normal subjects and children with cystic fibrosis. Clin Chim Acta 35:121–131. doi:10.1016/0009-8981(71)90302-0 5126986

[28] Van der Heiden C, Wadman SK, Ketting D, De Bree PK. 1971. Urinary and faecal excretion of metabolites of tyrosine and phenylalanine in a patient with cystic fibrosis and severely impaired amino acid absorption. Clin Chim Acta 31:133–141. doi:10.1016/0009-8981(71)90370-6 5101380

[29] Alkasir R, Ma Y, Liu F, Li J, Lv N, Xue Y, Hu Y, Zhu B. 2018. Characterization and transcriptome analysis of Acinetobacter baumannii persister cells. Microb Drug Resist 24:1466–1474. doi:10.1089/mdr.2017.0341 29902105

[30] Kashyap S, Sharma P, Capalash N. 2021. Potential genes associated with survival of Acinetobacter baumannii under ciprofloxacin stress. Microbes Infect 23:104844. doi:10.1016/j.micinf.2021.104844 34098109

[31] Hooppaw AJ, McGuffey JC, Di Venanzio G, Ortiz-Marquez JC, Weber BS, Lightly TJ, van Opijnen T, Scott NE, Cardona ST, Feldman MF. 2022. The phenylacetic acid catabolic pathway regulates antibiotic and oxidative stress responses in Acinetobacter. mBio 13:e0186321. doi:10.1128/mbio.01863-21 35467424 PMC9239106

[32] Lightly TJ, Frejuk KL, Groleau M-C, Chiarelli LR, Ras C, Buroni S, Déziel E, Sorensen JL, Cardona ST. 2019. Phenylacetyl coenzyme a, not phenylacetic acid, attenuates cepir-regulated virulence in Burkholderia cenocepacia. Appl Environ Microbiol 85:e01594-19. doi:10.1128/AEM.01594-19 31585996 PMC6881814

[33] Pribytkova T, Lightly TJ, Kumar B, Bernier SP, Sorensen JL, Surette MG, Cardona ST. 2014. The attenuated virulence of a Burkholderia cenocepacia paaABCDE mutant is due to inhibition of quorum sensing by release of phenylacetic acid. Mol Microbiol 94:522–536. doi:10.1111/mmi.12771 25155974

[34] Cerqueira GM, Kostoulias X, Khoo C, Aibinu I, Qu Y, Traven A, Peleg AY. 2014. A global virulence regulator in Acinetobacter baumannii and its control of the phenylacetic acid catabolic pathway. J Infect Dis 210:46–55. doi:10.1093/infdis/jiu024 24431277

[35] Green ER, Juttukonda LJ, Skaar EP. 2020. The manganese-responsive transcriptional regulator MumR protects Acinetobacter baumannii from oxidative stress. Infect Immun 88:e00762-19. doi:10.1128/IAI.00762-19 31792075 PMC7035938

[36] Jiao M, He W, Ouyang Z, Shi Q, Wen Y. 2022. Progress in structural and functional study of the bacterial phenylacetic acid catabolic pathway, its role in pathogenicity and antibiotic resistance. Front Microbiol 13:964019. doi:10.3389/fmicb.2022.964019 36160191 PMC9493321

[37] Mayrand D. 1979. Identification of clinical isolates of selected species of Bacteroides: production of phenylacetic acid. Can J Microbiol 25:927–928. doi:10.1139/m79-138 526889

[38] Loke WM, Jenner AM, Proudfoot JM, McKinley AJ, Hodgson JM, Halliwell B, Croft KD. 2009. A metabolite profiling approach to identify biomarkers of flavonoid intake in humans. J Nutr 139:2309–2314. doi:10.3945/jn.109.113613 19812218

[39] Marahatta A, Bhandary B, Lee M-R, Kim D-S, Lee YC, Kim S-R, Kim H-R, Chae H-J. 2012. Determination of phenylbutyric acid and its metabolite phenylacetic acid in different tissues of mouse by liquid chromatography with tandem mass spectrometry and its application in drug tissue distribution. J Chromatogr B Analyt Technol Biomed Life Sci 903:118–125. doi:10.1016/j.jchromb.2012.07.004 22841743

[40] Zaher A, ElSaygh J, Elsori D, ElSaygh H, Sanni A. 2021. A review of Trikafta: triple cystic fibrosis transmembrane conductance regulator (CFTR) modulator therapy. Cureus 13:e16144. doi:10.7759/cureus.16144 34268058 PMC8266292

[41] Commins N, Sullivan M, McGowen K, Koch E, Rubin E, Farhat M. 2022. Mutation rates and adaptive variation among the clinically dominant clusters of Mycobacterium abscessus. bioRxiv. doi:10.21203/rs.3.rs-1620528/v1 PMC10235944 37216535

[42] Yang Y, Xu G, Liang J, He Y, Xiong L, Li H, Bartlett D, Deng Z, Wang Z, Xiao X. 2017. DNA backbone sulfur-modification expands microbial growth range under multiple stresses by its anti-oxidation function. Sci Rep 7:3516. doi:10.1038/s41598-017-02445-1 28615635 PMC5471199

[43] Maitra R, Hamilton JW. 2005. Arsenite regulates cystic fibrosis transmembrane conductance regulator and P-glycoprotein: evidence of pathway independence. Cell Physiol Biochem 16:109–118. doi:10.1159/000087737 16121039

[44] Kelly SM, Vanslyke JK, Musil LS. 2007. Regulation of ubiquitin-proteasome system mediated degradation by cytosolic stress. Mol Biol Cell 18:4279–4291. doi:10.1091/mbc.e07-05-0487 17699585 PMC2043544

[45] Kozul CD, Hampton TH, Davey JC, Gosse JA, Nomikos AP, Eisenhauer PL, Weiss DJ, Thorpe JE, Ihnat MA, Hamilton JW. 2009. Chronic exposure to arsenic in the drinking water alters the expression of immune response genes in mouse lung. Environ Health Perspect 117:1108–1115. doi:10.1289/ehp.0800199 19654921 PMC2717138

[46] Mazumdar M, Christiani DC, Biswas SK, Ibne-Hasan OS, Kapur K, Hug C. 2015. Elevated sweat chloride levels due to arsenic toxicity. N Engl J Med 372:582–584. doi:10.1056/NEJMc1413312 25651269 PMC4368195

[47] Brynildsrud O, Bohlin J, Scheffer L, Eldholm V. 2016. Erratum to: rapid scoring of genes in microbial pan-genome-wide association studies with Scoary. Genome Biol 17:262. doi:10.1186/s13059-016-1132-8 27993146 PMC5165719

[48] Sherman BT, Hao M, Qiu J, Jiao X, Baseler MW, Lane HC, Imamichi T, Chang W. 2022. DAVID: a web server for functional enrichment analysis and functional annotation of gene lists (2021 update). Nucleic Acids Res 50:W216–W221. doi:10.1093/nar/gkac194 35325185 PMC9252805

[49] de Jong A, Kuipers OP, Kok J. 2022. FUNAGE-Pro: comprehensive web server for gene set enrichment analysis of prokaryotes. Nucleic Acids Res 50:W330–W336. doi:10.1093/nar/gkac441 35641095 PMC9252808

[50] Arndt D, Grant JR, Marcu A, Sajed T, Pon A, Liang Y, Wishart DS. 2016. PHASTER: a better, faster version of the PHAST phage search tool. Nucleic Acids Res 44:W16–W21. doi:10.1093/nar/gkw387 27141966 PMC4987931

