# Supplementary material for: Phenylacetic acid metabolic genes are associated with Mycobacteroides abscessus dominant circulating clone 1
Source: Microbiol Spectr. 2024 Sep 24;12(11):e01330-24. doi: 10.1128/spectrum.01330-24 (PMC11537035; doi:10.1128/spectrum.01330-24)
Supplement: Figures S1 to S3 — Fig. S1: Emory MAB population and Clinical Isolate Collection. Fig. S2: MAB DCC and Morphotype Colonization and Association with Lung Function. Fig. S3: paa Expression across publicly-available transcriptomes. [file spectrum.01330-24-s0001.docx]

**Supplemental Figures: Phenylacetic acid degradation is associated with *Mycobacteroides abscessus* dominate clonal complex 1 and supports growth**

Brittany N. Ross^1^, Emma Evans^1^, Marvin Whiteley^1,2^


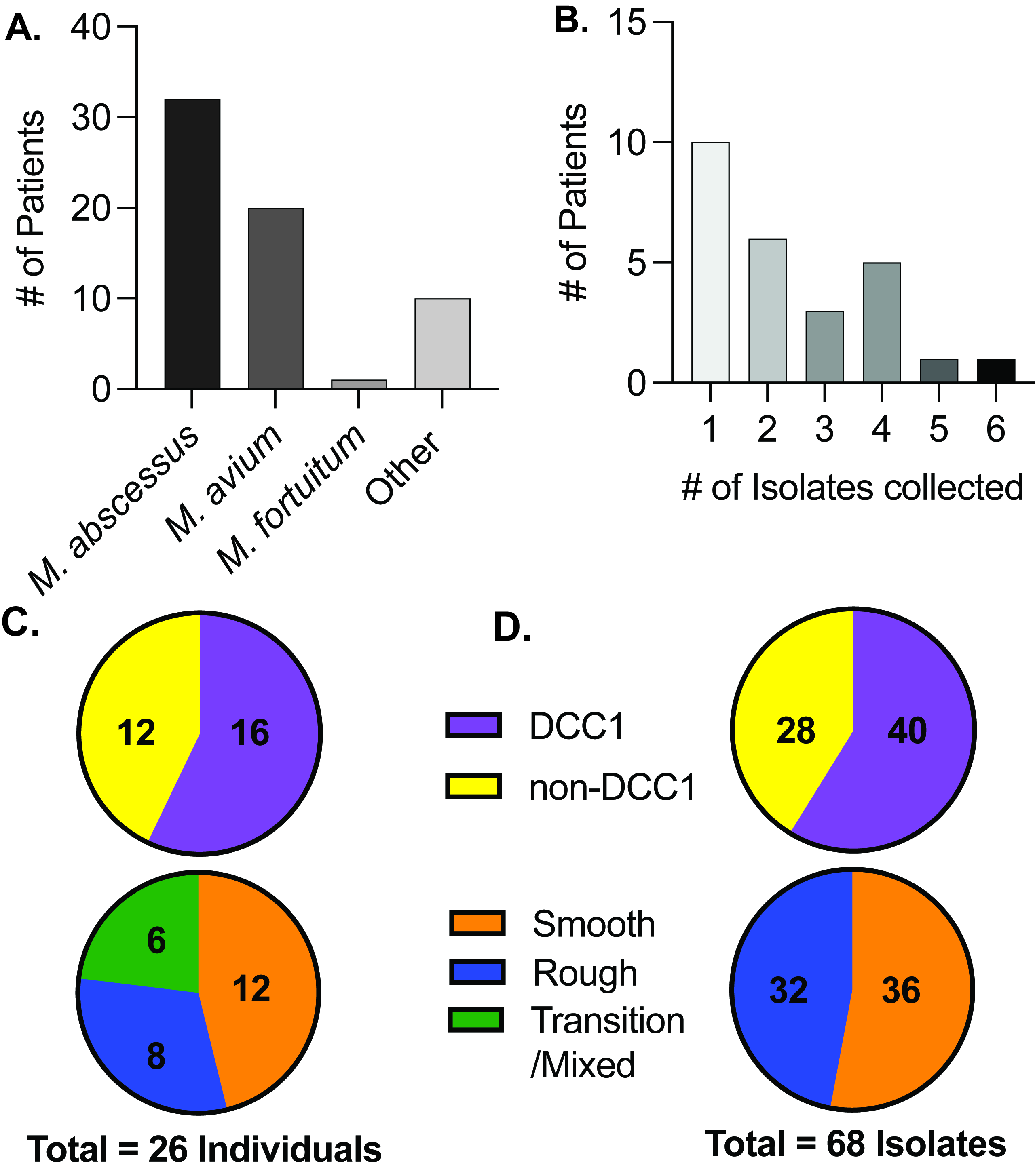


**Supplemental Figure 1. Emory MAB population and Clinical Isolate Collection.** **(A)** The distribution of participants infected with MAB compared to other Non-tuberculous Mycobacterium (NTMs) during the study timeframe. Of the 32 individuals’ continually culture positive for MAB, isolates were collected from 26 (81%) at multiple times between 2019-2022. **(B)** The number of isolates collected per participants was quantified. Isolates were characterized by DCC1 clustering and morphotyped for **(C)** each participant and **(D)** each isolate.


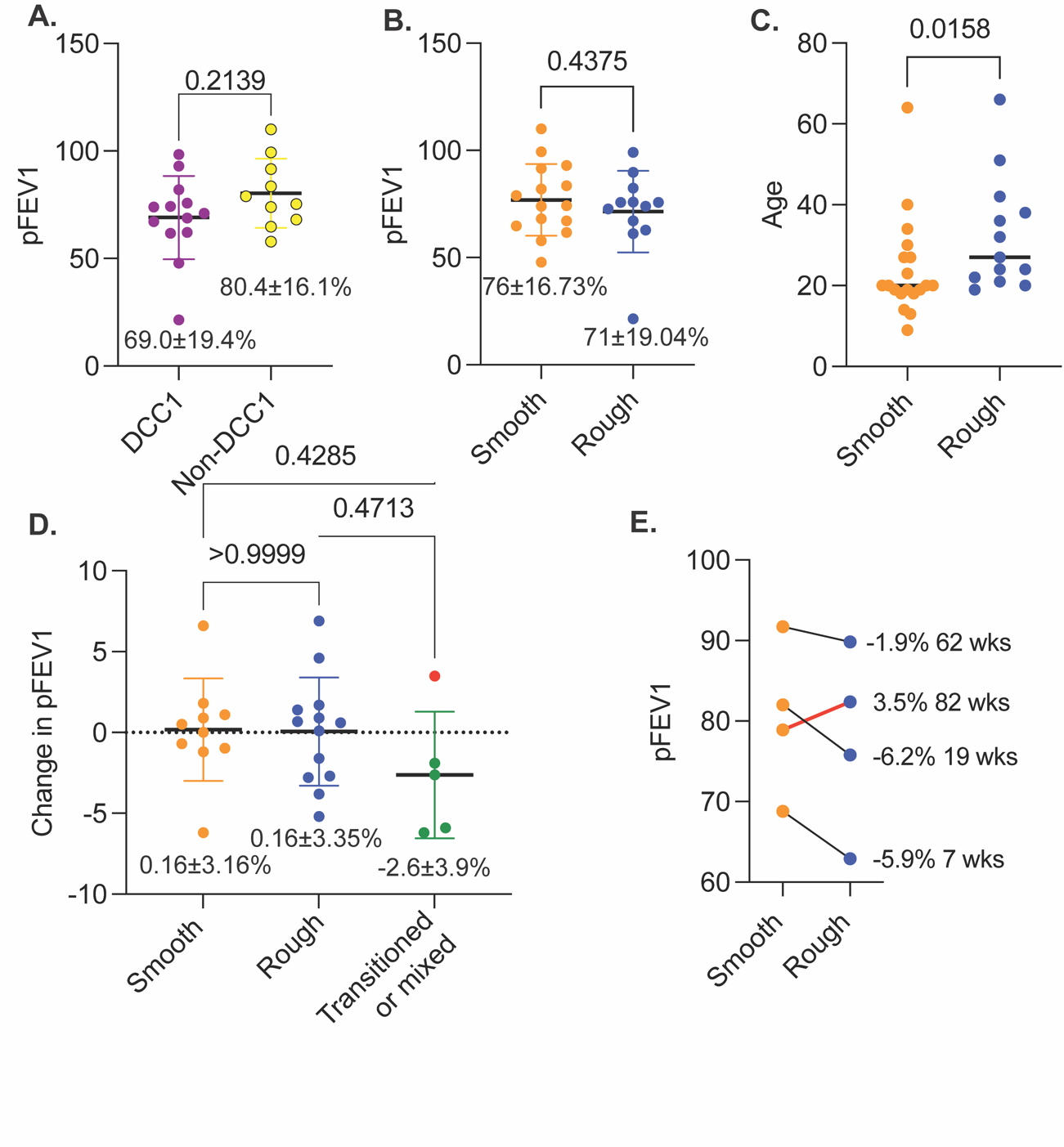


**Supplemental Figure 2. MAB DCC and Morphotype Colonization and Association with Lung Function.** Lung function is used to determine disease progression and represents the amount of air that can be expelled in one second compared to what is expected of a health individual (percent forced expiratory volume or pFEV1 is plotted. To examine the impact on isolate type on lung function pFEV1 was plotted by **(A)** DCC1 or non-DCC1 and **(B)** smooth or rough morphotype. **(C)** Since transition of morphotype may not occur for years to decades age was plotted by morphotype colonization. **(D)** To evaluate the impact of morphotype transition change in pFEV1 between timepoints for continuous smooth colonization, continuous rough, or individuals concurrently colonized or transition from smooth to rough colonization. **(E)** The pFEV1 was also plotted before and after the morphotype changed. The red line/datapoint in (D and E) the initiation of Trikafta. Note there is additional datapoint in (D) transition group compared to (E) which represents a participant that did not transition but was continually colonized by both smooth and rough in this study. Inlayed numbers represent averages ± standard deviations and p-value denoted above.

**
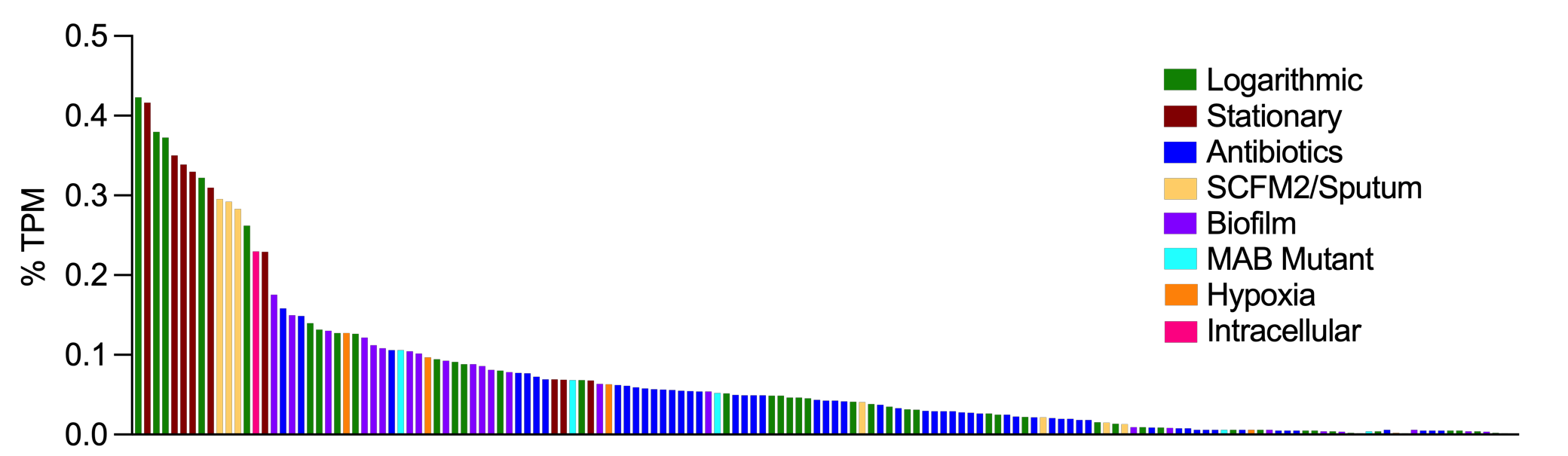
**

**Supplemental Figure 3. *paa* Expression across publicly-available transcriptomes.** Publicly-available MAB transcriptomes were downloaded and remapped with bowtie2. Reads were converted to transcripts per million (TPM), summed across the main enzymatic operon MAB_0902-0912, and presented as percent TPMs in ranked ordered. Only samples with reads mapping to 9 or more of the 11 genes were included. Different conditions are denoted by color.
